# Old player, new roles: defining the role of the plastidial phosphorylase

**DOI:** 10.1111/nph.70308

**Published:** 2025-06-18

**Authors:** David Seung, Slawomir Orzechowski, Joerg Fettke

**Affiliations:** ^1^ John Innes Centre Norwich Research Park Norwich NR4 7UH UK; ^2^ Biochemistry and Microbiology Institute of Biology SGGW, 159 Nowoursynowska Street Warsaw 02‐776 Poland; ^3^ Biopolymer Analytics, Institute of Biology and Biochemistry University of Potsdam Karl‐Liebknechtstr. 24/25 Potsdam 14476 Germany

**Keywords:** glucan phosphorylase, Pho1, Phs1, plastidial phosphorylase, starch, starch initiation, starch metabolism

## Abstract

The plastidial phosphorylase (Pho1 or Phs1; E.C. 2.4.1.1) is a ubiquitous enzyme among plants that catalyzes the formation and degradation of glucans. Although the first report connecting Pho1 with starch metabolism came out > 80 years ago, its precise role is still a matter of debate. In this article, we evaluate the catalytic and regulatory mechanisms of Pho1 in the context of known mechanisms in its animal, fungal, and bacteria homologs. We further discuss recent breakthroughs in understanding Pho1's function in initiating starch granule formation. This role is relevant to both photosynthetic and nonphotosynthetic tissues, as loss of Pho1 affects the regulation of the number of transitory starch granules in Arabidopsis leaves under various metabolic contexts, as well as the number of storage starch granules and/or starch granule morphology in wheat endosperm and potato tubers. Our comparison of phosphorylases across kingdoms reveals several regulatory mechanisms that require further investigation in plants. We also discuss emerging research on Pho1 protein interactions that give rise to other metabolic processes, such as photosynthesis. Overall, these multiple emerging roles of phosphorylase emphasize its importance in plant metabolism and its broad potential as a target for crop improvement.

## Introduction

Carbohydrates play a central role in the primary metabolism of living organisms, including the formation and degradation of complex polysaccharides (oligosaccharides, glycans, or glucans). Among the enzymes involved in these processes, phosphorylase is a ubiquitous representative in almost all kingdoms of life, including animals, plants, fungi, and bacteria (Hwang *et al*., 2020).

Phosphorylases (E.C. 2.4.1.1) catalyze the reversible transfer of glucosyl units from glucose 1‐phosphate (Glc‐1‐P) to the nonreducing end of an oligo‐ or polysaccharide, forming an α‐1,4 glycosidic linkage and releasing inorganic phosphate. It seems that oligo‐ or polysaccharides do not need to contain only α‐1,4 linkages, or consist solely of glucose, to act as the glucosyl acceptor, but they must have at least a length of degree of polymerization of 3 (Fettke *et al*., 2004, 2005a,b; Hwang *et al*., 2010). However, there are some exceptions proposed in the literature, including the synthesis of *de novo* glucans from Glc‐1‐P alone without acceptors (Cuesta‐Seijo *et al*., 2017; Nakamura *et al*., 2017). However, this is likely due to trace amounts of oligo‐ or polysaccharides present in the assays, as all described phosphorylases have a high binding affinity for them, and even tiny amounts are sufficient to initiate the synthesizing reaction. The reaction direction, toward synthesis or degradation, varies depending on the ratio of Glc‐1‐P to inorganic phosphate. There can be shifts in these ratios in the course of metabolism, and a subsequent temporal shift in the reaction direction (Fettke *et al*., 2010, 2012).

Plants and algae, in contrast to all other organisms, have two spatially separated isozymes of phosphorylase, a plastidial and a cytosolic isozyme (Steup, 1988). The cytosolic isozyme (designated as Pho2 or in *Arabidopsis thaliana* as Phs2) has a high affinity for highly branched, complex polysaccharides such as glycogen and heteroglycans, and is involved in the cytosolic processing of maltose from starch degradation (Fettke *et al*., 2004, 2005a,b, 2006; Lu *et al*., 2006). The cytosolic phosphorylase can also transfer glucose units onto acceptor substrates with sugars other than glucose at the nonreducing end (Fettke *et al*., 2005a,b). Potato mutants with altered expression of the cytosolic phosphorylase revealed structural alterations in these cytosolic heteroglycans (Fettke *et al*., 2005b). There is also evidence that Phs2 is involved in the regulation of carbohydrate metabolism, especially under low‐light and cold conditions in Arabidopsis (Schopper *et al*., 2014).

However, compared to Phs2, much more information is emerging on the important role of the plastidial isozyme (designated as Pho1 or in wheat and *Arabidopsis thaliana* as Phs1), which will be the focus of the review. Pho1/Phs1 resides in the plastid compartment where most of starch metabolism takes place, and resembles the properties of the maltodextrin phosphorylase of *Escherichia coli*, with a preference for short and linear glucans (Palm *et al*., 1987; Steup, 1990). Thus, this isozyme is similar to those found in glycogen‐metabolizing animals and bacteria. It is unknown whether the plastidial isoform can also act on more heterogeneous glycans, as shown for cytosolic phosphorylase (Fettke *et al*., 2004, 2005a,b).

Some plants and algae have more than one Pho1‐type isozyme, including potato (Sonnewald *et al*., 1995), *Lotus japonicus* (Qin *et al*., 2016), and *Chlamydomonas reinhardtii* (Dauvillée *et al*., 2006). Moreover, the affinity for various substrates can differ, allowing for further distinction of the isozymes (Eckermann *et al*., 2002).

**Fig. 1:**
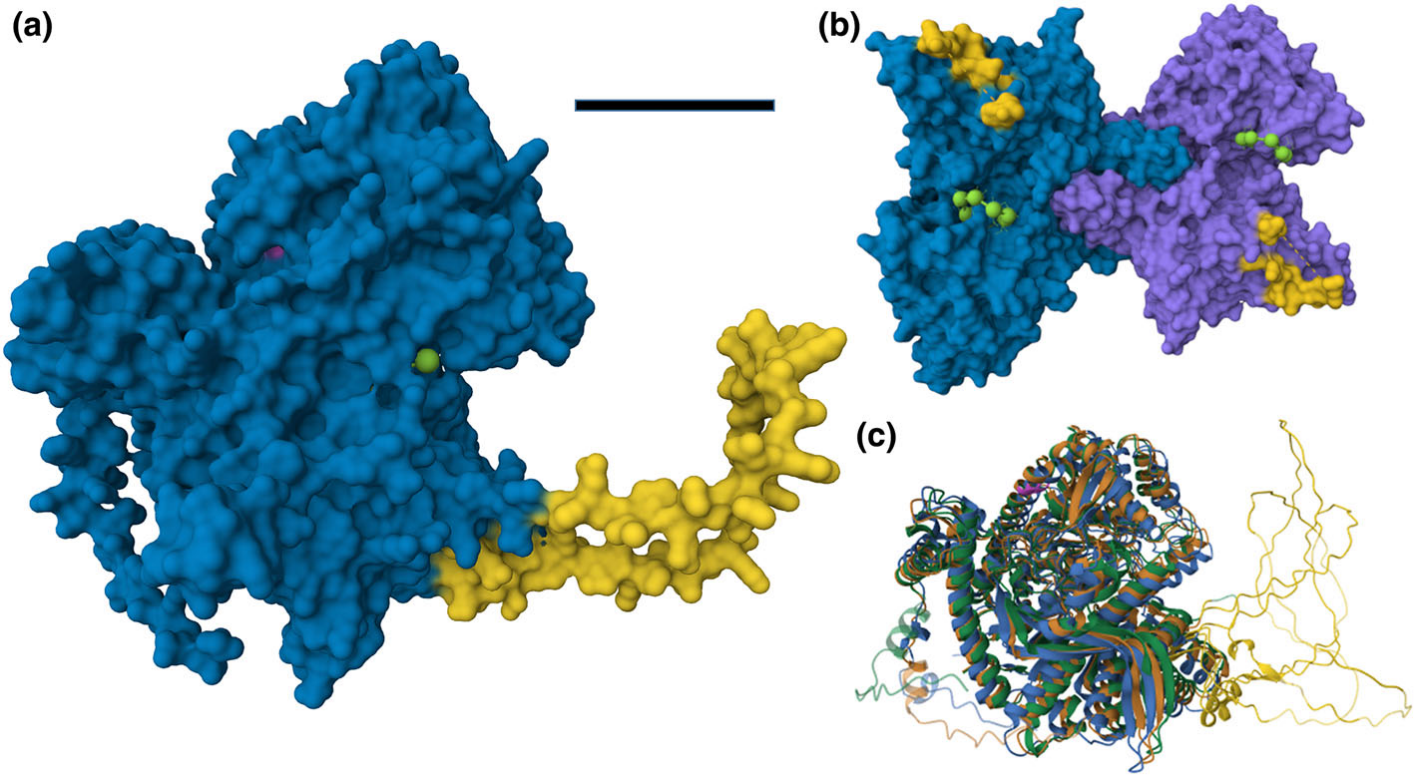
Structure of the plastidial phosphorylase. (a) Monomer of *Arabidopsis thaliana* Phs1. Bar, 2.5 nm. (b) Dimerization of potato Pho1a. (c) Overlay of Pho1/Phs1 from potato (*Solanum tuberosum* L., blue), wheat (*Triticum aestivum*, green), and Arabidopsis (*A. thaliana*, orange). Yellow, the L80 peptide; green, maltotetraose bound to the plastidial phosphorylase in (a, b); cyan, the catalytic lysine.

The plastidial isozyme of higher plants is further characterized by an additional region named L80, which is 50–82 amino acids in length depending on species, and in the middle of the primary sequence. This region is missing from Pho1 of algae, like *C. reinhardtii* (Dauvillée *et al*., 2006). L80 regions from different phosphorylases have low sequence similarity with each other, but are rich in serine and threonine, which can be phosphorylated. Initial structural modeling revealed that L80 forms a loop with an undefined structure (Hwang *et al*., 2016a). This is confirmed with the latest Alphafold3 models, where Pho1 from Arabidopsis, potato, and wheat all have L80 loops that protrude from the main enzyme domains (Fig. 1). The L80 region is not strictly required for catalytic activity (Hwang *et al*., 2016b), but is reported to influence enzyme biochemistry by influencing acceptor glucan binding (Mori *et al*., 1993a,b; Cuesta‐Seijo *et al*., 2017). It is also proposed to selectively mediate interactions with different proteins in response to metabolic alterations, including with Photosystem I in rice (Hwang *et al*., 2020; Koper *et al*., 2021, see below).

Almost all phosphorylases studied so far form dimers composed of two subunits. Most plant phosphorylases exist as homodimers (Fig. 1); however, there are examples of heterodimer formation, particularly in plants that contain more than one isozyme within the same cellular compartment. For example, potato has two plastidial isozymes, Pho1a and Pho1b, where Pho1a is abundant in leaves and Pho1b is undetectable in potato tubers. This results in homodimer and Pho1a/Pho1b heterodimer in leaves and flowers, and Pho1a homodimers in tubers (Albrecht *et al*., 1998, 2001). All phosphorylases also carry the pyridoxal 5' phosphate (PLP) cofactor in each subunit. PLP is linked by Schiff base formation to a specific lysine residue in the catalytic domain (Ariki & Fukui, 1975; Fukui *et al*., 1982; Newgard *et al*., 1989; Palm *et al*., 1990). It is essential for catalysis as it acts as a proton donor.

## Comparison of phosphorylase regulation from various regni

The widespread occurrence of phosphorylases provides a rich source of diversity in which different reaction and regulatory mechanisms can be found (Table 1). Comparing the enzymes across the kingdoms of life can demonstrate how diverse mechanisms have evolved to reflect different biological functions, while also pointing to possible common mechanisms that are largely unknown in plants and require further investigation.

**Table 1:** Regulation of phosphorylase activity in different organisms.

Origin	Allosteric effectors		Effect on Pho activity		References
	R‐form	T‐form	Activation	Inhibition	
Bacterial glycogen phosphorylase (*E. coli*)	+AMP Phospho‐histidine carrier protein (HPr)			+ADP‐Glc	Chen & Segel (1968); Seok *et al*. (1997)
Archea MmGP (*Methanococcales maripaludis*)			+Fru‐6‐P, +FBP	+ADP, +GMP, +ATP, +ADP‐Glc, +UDP‐Glc, +PEP, +citrate, PP_i_	González‐Ordenes *et al*. (2024)
Yeast glycogen phosphorylase 1		+Glc‐6‐P	Phosphorylation		Lin *et al*. (1997)
Plant plastidial phosphorylase *Chlorella vulgaris* Pea Maize			Phosphorylation	+ADP‐Glc +ADP‐Glc	Kruger & Ap Rees (1983); Nakamura & Imamura (1983); Grimaud *et al*. (2008)
Human glycogen phosphorylase mGP and bGP lGP	+AMP	+ATP +Glc‐6‐P +glucose	Phosphorylation Phosphorylation	Dephosphorylation +Glc‐1‐P +P_i_ Dephosphorylation	Lowry *et al*. (1964); Barford & Johnson (1989); Newgard *et al*. (1989); Barford *et al*. (1991); Crerar *et al*. (1995); Mathieu *et al*. (2016b, 2019)

AMP, adenosine monophosphate.

Similar to plants, several phosphorylase isozymes are present in animals. They are located in the same subcellular compartment (cytosol), but are active in different tissues and organs. There are three glycogen phosphorylase (GP) isozymes in human tissues—brain‐type glycogen phosphorylase (bGP), liver‐type glycogen phosphorylase (lGP), and muscle‐type glycogen phosphorylase (mGP)—which differ in function (Newgard *et al*., 1989). The sequences of these GP isozymes are highly conserved, with a sequence identity between 80 and 85% and very similar 3D structures (Lebo *et al*., 1984; Newgard *et al*., 1987, 1988, 1989; Adeva‐Andany *et al*., 2016). However, despite their high sequence identity, these three GP isozymes have distinct roles, as well as regulatory and functional properties. In the brain, both muscle and brain GP isoforms are present, in contrast to liver and muscle, where only one isoform predominates (Newgard *et al*., 1989; Pfeiffer‐Guglielmi *et al*., 2003; Saez *et al*., 2014). mGP is mainly present in skeletal muscle, where it rapidly breaks down muscle glycogen and is also involved in the production of adenosine triphosphate (ATP), which provides the necessary energy for muscle cell movement (Ørtenblad *et al*., 2013; Müller *et al*., 2015; Yang *et al*., 2024). lGP is mainly active in the liver, catalyzing the breakdown of glycogen to keep blood glucose levels stable (Proux & Dreyfus, 1973; Agius, 2015; Müller *et al*., 2015). The low glycogen content in the brain, in turn, suggests that it is exclusively an emergency reserve mobilized by bGP and mGP under ischemic conditions (Sagar *et al*., 1987).

There is more information on the regulation of phosphorylases in animals, compared to plants (Table 1). GP activity is regulated in response to energy requirements. GP in the liver is regulated by insulin and glucagon to control blood glucose levels (van de Werve *et al*., 1977; Agius, 2015). During prolonged physical activity, membrane depolarization and noradrenaline release in muscles stimulate the glycogenolytic cascade and thus supply the muscle cells with energy (Jensen & Richter, 2012). The mobilization of glycogen during brain activity is facilitated by extracellular signals, via neurotransmitters and neuromodulators such as noradrenaline, dopamine, histamine, and serotonin (Hutchins & Rogers, 1970; Magistretti *et al*., 1981; Sorg & Magistretti, 1991, 1992; Allaman *et al*., 2003; Xu *et al*., 2013, 2014; Coggan *et al*., 2018). Allosteric activation and phosphorylation are the two main regulatory strategies controlling phosphorylase activity, and the two strategies can occur simultaneously.

All three human GP isoforms are allosterically regulated (Newgard *et al*., 1989; Roach, 2002). GP has multiple allosteric sites that can, at the same time, bind allosteric effectors with activating and inhibitory effects. This creates several intermediate activation states that allow precise control of enzyme activity and thus accurate control of energy requirements (Newgard *et al*., 1989; Barford *et al*., 1991; Sprang *et al*., 1991; Oikonomakos *et al*., 1995; Lukacs *et al*., 2006). An increased energy demand, characterized by a high adenosine monophosphate (AMP): ATP ratio, leads to preferential binding of AMP at its allosteric site. This leads to the transition or stabilization of GP in its active R‐state (Barford *et al*., 1991; Sprang *et al*., 1991). The enzyme can then degrade glycogen to Glc‐1‐P, which is further used in glycolysis and the tricarboxylic acid (TCA) cycle to produce ATP. On the other hand, high levels of ATP or glucose 6‐phosphate (Glc‐6‐P) reflect a high cellular energy state. Both ATP and Glc‐6‐P can bind to the same allosteric site as AMP, resulting in inhibition of glycogen phosphorolysis via inactivation of the enzyme (Newgard *et al*., 1989). Similarly, glucose binding to the catalytic site of GP results in the transition to, or stabilization in, the T‐state. In addition to their allosteric effects, glucose and Glc‐1‐P inhibit GP activity by competing with glycogen for binding to the catalytic site (Newgard *et al*., 1989). Although the human GP isoforms are similar in sequence, they differ considerably in their allosteric regulation. For example, bGP and mGP bind AMP differently: the binding of AMP to mGP is cooperative (i.e. the binding of AMP to one mGP monomer leads to activation of the other monomer), whereas the binding of AMP to bGP is noncooperative (Crerar *et al*., 1995; Mathieu *et al*., 2016b, 2019). By contrast, lGP is less susceptible to allosteric regulation and is controlled almost exclusively by phosphorylation (Lowry *et al*., 1964; Newgard *et al*., 1989). Theoretically, GP can also catalyze the reaction of glycogen synthesis from Glc‐1‐P *in vitro*, but *in vivo*, this reaction does not occur because the intracellular ratio of inorganic phosphate to Glc‐1‐P is high, thus resulting in glycogenolysis (Newgard *et al*., 1989).

In addition to allosteric regulation, lGP is activated by reversible phosphorylation at Serine 14 by a specific phosphorylase kinase. This is triggered by a glycogenolytic cascade that is activated by the binding of hormones (such as glucagon or noradrenaline) to their receptors, which activates the production of cyclic AMP (cAMP) by adenylyl cyclase and the subsequent activation of protein kinase A (PKA), which eventually leads to the activation of phosphorylase kinase. Other factors that cause the release of calcium from the endoplasmic reticulum can also activate the glycogenolytic cascade through the direct interaction with phosphorylase kinase and adenylyl cyclase (Heilmeyer, 1991). Dephosphorylation of GP is facilitated by protein phosphatase 1 (PP1), and the dephosphorylated form of GP is inactive in the absence of an allosteric activator (Barford & Johnson, 1989; Newgard *et al*., 1989; Barford *et al*., 1991).

Regulation by metabolites and phosphorylation may be a common feature in both plant and animal phosphorylases. In the degradation direction, the pea plastidial phosphorylase is inhibited by ADP‐glucose (ADP‐Glc) *in vitro* (Matheson & Richardson, 1978; Kruger & Ap Rees, 1983). Similar observations were made for the *Chlorella* phosphorylase, where inhibition by ADP‐Glc was observed in both synthesis and degradation directions (Nakamura & Imamura, 1983). Phosphorylation of the plastidial phosphorylase has been reported in plants, either for the activation of the enzyme or for regulating interactions with other proteins (Tetlow *et al*., 2004; Grimaud *et al*., 2008). Proteomic studies in maize revealed that some serine or threonine residues in L80 are phosphorylated: *T*_537_, *S*_547_, *S*_556_, *S*_565_, and *S*_566_ (Walley *et al*., 2013). Similarly, it was shown that a serine residue in L80 of Pho1 from sweet potato roots was phosphorylated, but no alteration in activity was detectable (Young *et al*., 2006). Thus, the exact implication of the phosphorylation needs further investigation.

Redox regulation may be another regulatory mechanism important for both plant and animal phosphorylases. Although mGP and lGP are not thought to be redox‐sensitive, bGP is very sensitive to reactive oxygen species (ROS) such as H_2_O_2_, a common oxidant present in the environment, leading to a reversible inhibition and redox regulation of glycogenolysis (Mathieu *et al*., 2016a, 2017). It involves the formation of an inhibitory intramolecular disulfide bond by the cysteine residues Cys318 and Cys326, which prevents allosteric activation by blocking the binding of AMP. This modification does not affect the regulation of bGP by phosphorylation (Mathieu *et al*., 2016a). Plants often deal with oxidative stress, especially in the chloroplast, and intracellular ROS are important signaling molecules. There are many examples of redox‐regulated starch metabolic enzymes, where inhibition of activity through disulfide bridge formation is reversed by reductive activation by thioredoxins (e.g. AGPase, BAM1, AMY3; Sparla *et al*., 2006; Hädrich *et al*., 2012; Seung *et al*., 2013). Although redox regulation in plant phosphorylases has not been fully explored, there is some evidence that it could be important. Proteomic studies proposed Pho1 of rice and wheat could be redox sensitive (Balmer *et al*., 2006; Xu *et al*., 2010). Further, Pho1 undergoes structural changes in response to redox conditions, including disulfide bridge formation (Subasinghe *et al*., 2014; Hwang *et al*., 2020). *In vitro* studies with barley Pho1, however, revealed no alteration of the activity by dithiothreitol or barley thioredoxins (Cuesta‐Seijo *et al*., 2017). However, it is possible that the redox regulation of plant phosphorylases is important for protein–protein interactions *in vivo*, and not necessarily for the regulation of activity. Thus, the redox regulation of plant phosphorylases is an area that requires further investigation.

When comparing animal and plant phosphorylase regulation, the yeast glycogen phosphorylase represents a somewhat ‘intermediate’ system. The glycogen phosphorylase, GPH1, mediates one pathway for glycogen degradation in yeast (Hwang *et al*., 1989). Expression of GPH1 is induced as cells approach the stationary phase, as is the case for many other genes involved in glycogen storage and/or utilization (Hwang *et al*., 1989). GPH1 is regulated by both phosphorylation and allosteric regulation. The unphosphorylated form is in the T state with low activity. Phosphorylation of GPH1 stabilizes the highly active R‐state, which exhibits a hyperbolic rate curve depending on Glc‐1‐P concentration. Glc‐6‐P allosterically inhibits the dephosphorylated enzyme in a noncooperative mechanism by stabilizing the T‐state, and cooperatively inhibits the phosphorylated enzyme by causing a transition from the R‐state to the T‐state (Lin *et al*., 1997). Glc‐6‐P inhibits the dephosphorylated enzyme more effectively than the phosphorylated form. Yeast and animal phosphorylases share a conserved Glc‐6‐P binding site, glucose‐binding amino acid residues, and glycogen‐binding site. However, yeast and animal enzymes differ in important kinetic properties. Yeast glycogen phosphorylase is neither activated by AMP nor inhibited by glucose at physiological concentrations (Sagardía *et al*., 1971; Tanabe *et al*., 1987). Furthermore, the site of the phosphorylation that activates activity is different, being at threonine 31 (Lin *et al*., 1995, 1996, 1997) rather than the serine phosphorylation in animal enzymes, and involves a different kinase (Lerch & Fischer, 1975; Wingender‐Drissen & Becker, 1983; Hwang & Fletterick, 1986). The dephosphorylated enzyme in the T‐state can be present as a dimer (glycogen‐bound) or a tetramer (nonglycogen‐bound). The dimer in the T‐state is a better substrate for the protein kinase than the tetramer, and phosphorylation causes the transition of the active dimer from the T‐state to the R‐state. The enzyme in this conformation can be converted to either the tetrameric T‐state (glycogen‐free) or the dimeric T‐state (glycogen‐bound) upon binding of Glc‐6‐P (Lin *et al*., 1995, 1997).

In contrast to the diversely regulated eukaryotic glycogen phosphorylases, bacterial glycogen phosphorylases (GlgP) have less prominent regulatory properties. It is assumed that they are mainly controlled at the level of gene expression (Newgard *et al*., 1989; Hudson *et al*., 1993; Watson *et al*., 1997; Schinzell & Nidetzky, 1999; Bonafonte *et al*., 2000), but they may also be subject to some post‐translational regulation. In the synthesis direction, the *E. coli* GlgP is moderately activated by AMP and inhibited by ADP‐Glc (Chen & Segel, 1968). However, in the degradative direction, GlgP does not exhibit regulation by physiologically relevant substrates such as AMP, ADP‐Glc, UDP‐glucose, glucose, and hexose phosphates (Alonso‐Casajús *et al*., 2006). The activity of GlgP can also be allosterically regulated in *E. coli* through its interaction with the phosphohistidine carrier protein (HPr) of the phosphotransferase system (Seok *et al*., 1997).

Interestingly, a unique GP from archaea *Methanococcales maripaludis* (*Mm*GP) has significant differences in catalytic mechanism and regulation compared to bacteria and eukaryotes. In contrast to all other characterized phosphorylases, *Mm*GP does not require PLP for activity. Compared to yeast glycogen phosphorylase (regulated by AMP and Glc‐6‐P), MmGP activity appears to be sensitive to metabolic intermediates, PEP, and FBP. Metabolites such as ADP‐Glc, UDP‐glucose, PPi, and ADP have been observed to play a regulatory role in individual cases of eukaryotic and bacterial enzymes. Most of these molecules have not yet been identified as factors that can influence glycogen phosphorylase activity in prokaryotes (González‐Ordenes *et al*., 2024).

## Metabolic role of the plastidial phosphorylase in starch initiation in photosynthetic tissues

The exact function of the plastidial phosphorylase in plants has been under debate for decades. Discussion on the direction of the reaction *in vivo* dates back as early as the 1940s (Hanes, 1940), and initial reports favored a role of the enzyme in the direction of degrading α‐glucans and starch into glucose phosphates—similar to the role of GP in animals and bacteria (Steup & Schächtele, 1981; Newgard *et al*., 1989). This assumption was supported by the higher concentration of inorganic phosphate compared to Glc‐1‐P in leaves and barley grains (Kruger & Ap Rees, 1983; Tiessen *et al*., 2012). However, the local and dynamic changes in metabolite concentrations were not taken into account, including the ratio of both inorganic phosphate and Glc‐1‐P in the chloroplast stroma and in the direct vicinity to the enzyme.

To date, there is no genetic evidence to support the role of the plastidial phosphorylase in starch degradation. However, there is strong evidence for the role of the enzyme in starch synthesis—highlighting a major difference to the GPs described above, which are almost exclusively involved in glycogen degradation. The first report of a role for Pho1 in starch synthesis was in the unicellular algae *C. reinhardtii*, where a mutant line lacking the plastidial phosphorylase Pho1 (here named PhoB) had reduced starch content, a higher proportion of amylose in starch, and abnormal morphology of starch granules (Dauvillée *et al*., 2006). Shortly after, rice mutants defective in Pho1 were reported to produce grains with a shrunken phenotype and low starch content, particularly when grown at low temperature (Satoh *et al*., 2008). This phenotype, together with the ability of Pho1 to elongate maltooligosaccharides *in vitro*, led to the proposal that the enzyme might be important in starch granule initiation, particularly at low temperatures (Satoh *et al*., 2008; Nakamura, 2015).

In recent years, we gained substantial evidence that Pho1 also plays an important role in granule initiation and the control of starch granule number in plant photosynthetic tissues. Originally, knockout mutants of Pho1 in potato and the common ‘Col‐0’ accession of *Arabidopsis thaliana* revealed no obvious alterations in growth or starch metabolism when grown under optimal growth conditions, leading to the suggestion that Pho1/Phs1 (potato/Arabidopsis) is dispensable in leaves (Sonnewald *et al*., 1995; Zeeman *et al*., 2004). We now know that a strong effect of Pho1/Phs1 knockout mutations on starch metabolism in leaves can be observed under certain conditions. Other accessions of Arabidopsis showed an altered starch amount and turnover following elimination of PHS1 under controlled conditions (data not published). Further, Arabidopsis *dpe2phs1* double mutants lacking the plastidial phosphorylase in addition to the disproportionating enzyme 2 (DPE2, EC: 2.4.1.25, Chia *et al*., 2004; Lu & Sharkey, 2004; Fettke *et al*., 2006) revealed strongly altered starch metabolism, with a striking reduction in the starch granules number per chloroplast. In wild‐type Arabidopsis, the typical number of starch granules per chloroplast is mainly 5–6 (Li *et al*., 2022), but *dpe2phs1* only has one or no granules per chloroplast (Malinova *et al*., 2014). Extensive analyses revealed that young leaves mostly contain one starch granule per chloroplast, whereas mature leaves were starch‐free (Malinova *et al*., 2014; Li *et al*., 2022). Interestingly, the number of starch granules was not fixed over development in the double mutants, revealing three distinct phases of starch metabolism. The first or prior phase is characterized by similar starch metabolism as the wild‐type, including a comparable number of starch granules per chloroplast, followed by a phase with reduced granule number, reflecting the above‐mentioned phenotype. However, and most interestingly, this condition is followed by a recovery phase, during which granule number increases to those similar to the wild‐type (Li *et al*., 2022). Thus, the metabolic regulation of the starch granule number per chloroplast is affected when the plastidial phosphorylase is lacking, and further, at least two different phases of starch granule initiation take place (Malinova *et al*., 2018; Li *et al*., 2022).

Another clear indication that plastidial phosphorylase has variable functions depending on the metabolic situation is the strong effect observed on the *dpe2phs1* phenotype after blocking starch degradation, regardless of whether this is achieved by growing under continuous light or by introducing mutations into the starch degradation pathway (Malinova & Fettke, 2017). The total block of starch degradation (through *gwd* and *pwd* mutations; for GWD and PWD, see Compart & Fettke, 2025) in the *dpe2phs1* background resulted in a granule number phenotype that is similar to the wild‐type, while the incomplete disturbance of the starch degradation in *dpe2phs1* again altered starch granule numbers (Muntaha *et al*., 2022). This suggests that carbon fluxes affect the regulation of starch granule number and that plastidial phosphorylase plays a role in this regulation. Thus, plastidial phosphorylase could be involved in increasing the starch granule number available for the carbon turnover in leaves, and thus, at least two different starch initiation events exist (Malinova *et al*., 2018; Mérida & Fettke, 2021).

The exact mechanism by which Phs1/Pho1 affects starch granule number in chloroplasts is not known. It is so far undisputed that the glucosyltransferase, starch synthase 4 (SS4; EC: 2.4.1.21; Roldán *et al*., 2007; Szydlowski *et al*., 2009) is essential for starch granule initiation in Arabidopsis leaves. Similar to *dpe2phs1*, the *ss4* mutant has strongly reduced starch content, and has mostly no starch granules or only one per chloroplast (Roldán *et al*., 2007; Malinova *et al*., 2017). Interestingly, the *dpe2phs1ss4* triple mutants still contained starch granules, demonstrating that the initiation of starch granules is, in principle, still possible when both SS4 and Phs1 are missing (Malinova *et al*., 2017). However, in contrast to *dpe2phs1*, the granule number in *dpe2phs1ss4* is less variable across leaves and development (Malinova *et al*., 2017). In addition to SS4 and Phs1, several other noncatalytic proteins are implicated in granule initiation in Arabidopsis chloroplasts, including PROTEIN TARGETING TO STARCH family members (PTST2 and PTST3), MAR1‐BINDING FILAMENT PROTEIN (MFP1), MYOSIN‐RELATED CHLOROPLAST PROTEIN (MRC) (Seung *et al*., 2017, 2018). These proteins are proposed to act by interacting with SS4 and influencing its substrate‐binding and/or localization (Sharma *et al*., 2024). Arabidopsis mutants deficient in any of these proteins have reduced granule numbers per chloroplast. Excitingly, *in vitro* experiments demonstrated protein–protein interaction between SS4 and Phs1, raising the possibility that Phs1 acts via indirect interactions also with the other granule initiation proteins (Malinova *et al*., 2018). Further work is required to discover whether it also interacts with other initiation proteins in Arabidopsis.

## Metabolic role of the plastidial phosphorylase in granule initiation in amyloplasts

In contrast to photosynthetic tissues, various protein–protein interactions of the plastidial phosphorylase with key starch biosynthesis enzymes have been reported in heterotrophic tissues during storage starch formation. In all cereal species examined, Pho1 is reported to interact with the major starch branching enzyme (SBE) isoform, although different configurations of the complex are reported between species. In maize, a Pho1, SBEIIb, and SSIIa configuration was observed (Liu *et al*., 2009); whereas Pho1 and SBEIIa were observed in rice (Crofts *et al*., 2015; Nakamura *et al*., 2017), and Pho1, SBEI, and SBEIIb in wheat (Tetlow *et al*., 2004). The interaction between the rice Pho1 and SBE synergistically enhances the synthesis of branched glucans *in vitro* and was proposed to be a key process required for starch granule initiation (Nakamura *et al*., 2017). Similar synergistic protein–protein interactions have been observed between the rice Pho1 and Disproportionating Enzyme 1 (DPE1; Hwang *et al*., 2016a). These interactions with biosynthetic enzymes strongly point toward a role for Pho1 in starch biosynthesis.

In addition to the earlier work in rice endosperm, recent years have seen more genetic evidence supporting a role for Pho1 in starch synthesis. In contrast to the conditional phenotype of rice, the loss of Phs1 in wheat was recently shown to greatly affect starch granule number within the grain under normal growth conditions (Kamble *et al*., 2023). Unlike most other cereals, wheat and other *Triticeae* (e.g. barley and rye) have a distinct temporal pattern of starch granule initiation during grain development, leading to a bimodal starch granule population in the endosperm. Large A‐type granules initiate during early grain development, while small B‐type granules initiate during mid‐grain development (15–20 d post‐anthesis) (Kamble *et al*., 2023). Mutants deficient in Phs1 have normal numbers of A‐type starch granules, but a dramatic reduction in the number of B‐type starch granules. This phenotype is consistent with Phs1 expression, which peaks during mid‐grain development in the endosperm. Phs1 interacts with the granule initiation protein B‐GRANULE CONTENT 1, which is the ortholog of PTST2 in wheat and is also essential for B‐type granule initiation (Chia *et al*., 2020). Phs1 and its interaction with granule initiation proteins have therefore gained a novel function in the *Triticeae* to mediate the initiation of the unique B‐type granules in these species. How its interaction with BGC1 is coordinated with the previously observed interactions with SBE is not yet known. Regulation of Phs1 activity in the wheat endosperm in response to environmental and metabolic fluctuations also remains to be investigated. For example, the relative amount of B‐type granules is known to be sensitive to environmental conditions, such as heat (Blumenthal *et al*., 1995) and water stress (Singh *et al*., 2008), but how this relates to effects on Phs1 activity is not known.

Similarly, recent analyses of Pho1 knockout mutants generated with CRISPR/Cas9 have implicated the enzyme in starch synthesis in potato tubers. Loss of Pho1 results in not only smaller, more rounded starch granules and more granules per tuber cell, but also lower starch content per tuber and reduced amylose content (Sharma *et al*., 2023). The extent to which these phenotypes are due to defects in starch granule initiation as opposed to defects in other aspects of starch synthesis remains to be determined. Notably, the interaction between Pho1 and PTST2 in wheat was not observed between the potato proteins (Hochmuth *et al*., 2024).

There is evidence for a more general involvement of Pho1 in starch and maltodextrin metabolism in tuber cells. Experiments with potato tuber slices revealed that exogenous Glc‐1‐P is taken up by the parenchyma cells and converted into starch. This conversion into starch is mainly driven by the plastidial phosphorylase (Fettke *et al*., 2010). Also, the plastidial phosphorylase is directly involved in the maltodextrin metabolism in amyloplasts, including their turnover and formation of longer maltodextrins (Flores‐Castellanos & Fettke, 2023). These experiments clearly showed strong metabolic flexibility, and it was further speculated that this pathway is particularly important under abiotic stress, particularly cold (Fettke *et al*., 2012). Interestingly, this is consistent with the importance of Pho1 under low‐temperature conditions observed in rice (Satoh *et al*., 2008; Hwang *et al*., 2016b). However, the relevant transporters responsible for Glc‐1‐P uptake are unknown in potato yet. One of the many sugar transporters can likely transport Glc‐1‐P, perhaps only with low specificity. In Arabidopsis, two candidates were identified (Malinova *et al*., 2020), but only a moderate flux of Glc‐1‐P toward starch was observed in incubation experiments with isolated Arabidopsis proto‐ and chloroplasts in the light (Fettke *et al*., 2011). However, in this system, starch synthesis was independent of the plastidial phosphorylase activity. It should be noted that ADP‐Glc formed during the light phase in leaf tissues is an inhibitor of phosphorylase activity and thus the pathway could be blocked in light conditions (Hwang *et al*., 2010).

## Additional functions of the plastidial phosphorylase

**Fig. 2:**
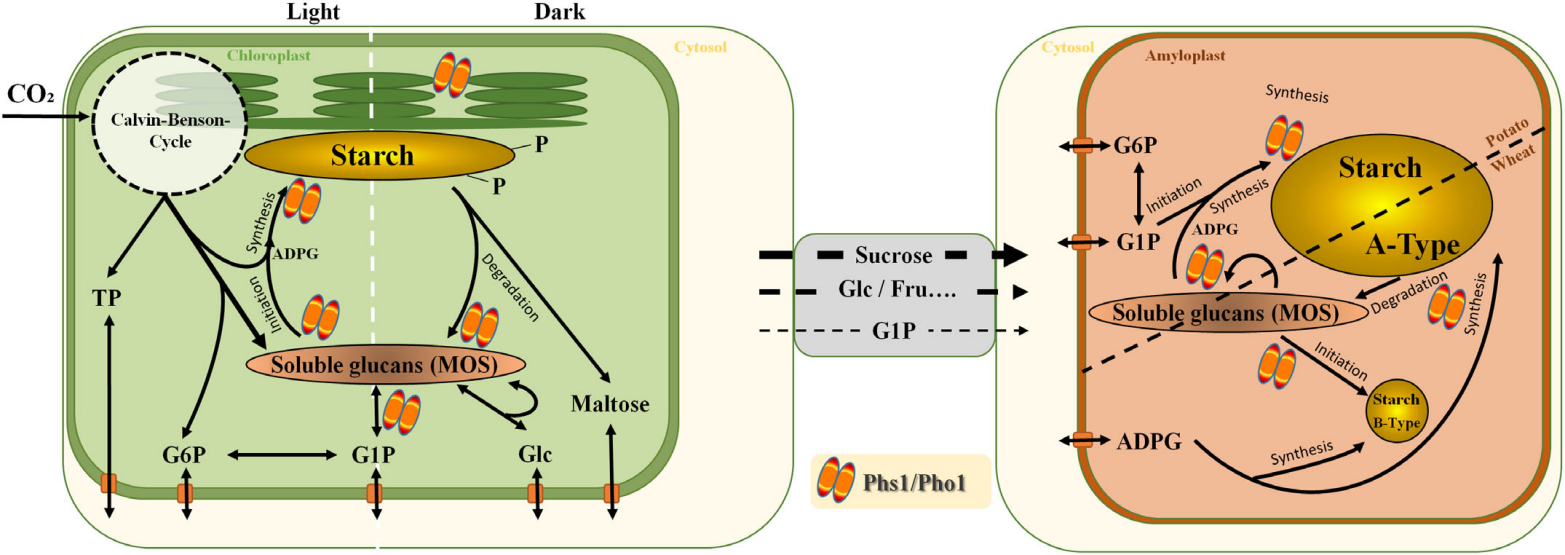
Processes involving the plastidial phosphorylase. The roles in metabolism in leaves (left) are mainly derived from information in Arabidopsis, whereas the roles in heterotrophic tissues focus on the potato tuber and wheat endosperm (right).

Over the last years, it has become clear that Phs1/Pho1 participates in an increasing number of metabolic situations, as summarized in Fig. 2. However, further functions are also emerging, including an interaction of Pho1 with PsaC of Photosystem I in *Oryza sativa* (Koper *et al*., 2021). Interestingly, the L80 domain seems to be involved in this interaction. Further, mutants lacking the L80 domain were reported to be more susceptible to cold. Similarly, the interaction of Phs1 and photosynthesis proteins was observed for *Arabidopsis thaliana* (Muntaha & Fettke, 2025). However, to what extent Pho1/Phs1 is able to modulate photosynthesis is unclear. Further, a significant role of Pho1 in potato leaves in response to low temperature and drought stress has been demonstrated (Orawetz *et al*., 2016; Orzechowski *et al*., 2021; Paprocka *et al*., 2024).

Given that plants have phosphorylases in both the plastid and cytosol, one could speculate that the enzymes also have an important role in integrating metabolism and signaling between compartments. Such mechanisms have been observed for other enzymes that occur across multiple compartments, like the malate valve mediated by malate dehydrogenases (Selinski & Scheibe, 2019). Typically, multiple plastids exist within a single cell, and an integration of processes in the various plastids to the cytosol seems essential in order to be able to control cellular processes effectively. In this context, the discovery of Glc‐1‐P transporters across the chloroplast membrane is even more interesting because they would further enhance such signal integration.

Such regulatory roles, combined with its metabolic roles, emphasize the great importance of phosphorylase. However, we must note that it is unclear whether all currently known functions are related to its activity. It could also be that some functions of phosphorylase are ‘moonlighting’ functions that are only determined by structure or protein interaction. Therefore, a precise elucidation of the regulation of plant enzymes is crucial for further understanding and should be the first step. Importantly, its multiple functions highlight the broad potential for crop improvement through this enzyme. In many crops such as wheat, rice, maize, and potato, alterations of the plastidial phosphorylase can contribute to customized starches and/or food security.

## Competing interests

None declared.

## Disclaimer

The New Phytologist Foundation remains neutral with regard to jurisdictional claims in maps and in any institutional affiliations.
